# Frugal innovation in medicine for low resource settings

**DOI:** 10.1186/s12916-016-0651-1

**Published:** 2016-07-07

**Authors:** Viet-Thi Tran, Philippe Ravaud

**Affiliations:** Department of General Medicine, Paris Diderot University, Paris, France; Centre de Recherche en Epidémiologie et Statistiques, INSERM U1153, Paris, France; Centre d’Épidémiologie Clinique, Hôpital Hôtel-Dieu, Assistance Publique-Hôpitaux de Paris, 1 place du Parvis Notre-Dame, Paris, 75181 France; Paris Descartes University, Paris, France; Department of Epidemiology, Columbia University Mailman School of Public Health, New York, NY USA

**Keywords:** Global health, Developing countries, Diffusion of innovation

## Abstract

Whilst it is clear that technology is crucial to advance healthcare: innovation in medicine is not just about high-tech tools, new procedures or genome discoveries. In constrained environments, healthcare providers often create unexpected solutions to provide adequate healthcare to patients. These inexpensive but effective frugal innovations may be imperfect, but they have the power to ensure that health is within reach of everyone. Frugal innovations are not limited to low-resource settings: ingenuous ideas can be adapted to offer simpler and disruptive alternatives to usual care all around the world, representing the concept of “reverse innovation”. In this article, we discuss the different types of frugal innovations, illustrated with examples from the literature, and argue for the need to give voice to this neglected type of innovation in medicine.

## Background

The story goes that Laennec, who was embarrassed to put his ear on the chest of a young woman, used a sheaf of paper rolled into a cylinder to auscultate the heart and thus invented the stethoscope [1]. Today, we would have qualified his ingenuity as “frugal innovation”. Frugal innovation is a broad term encompassing heterogeneous activities providing effective functional solutions to common problems encountered by “the many”, with a minimal use of resources [2]. Innovations frequently arise in low-resource settings, when usual solutions are too expensive or not available. In these constrained environments, people work with what they have, using affordable but effective tools, processes and techniques to solve their problems.

**Fig. 1:**
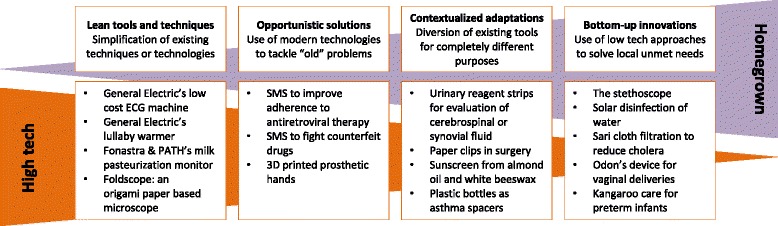
Examples of frugal innovations in healthcare. More examples and references are available on our website (http://frugal-innovation-medicine.com/)

Two forces drive frugal innovation and contribute to the development of these tools, processes and/or techniques. One is from companies or is supported by organizations such as the WHO [3] or PATH [4], the leader in global health innovation, to provide accessible technologies by simplifying existing high-tech tools. The other is from low-cost homegrown “fixes”, using low-tech (or even “no-tech”) solutions to solve unmet needs (Fig. 1).

## Sub-types of frugal innovation

To understand, define and help people identify frugal innovation, we propose to distinguish four subtypes of frugal innovation in medicine.

*Lean tools* and techniques refer to the simplification and adaptation of existing technologies to greatly reduce costs and provide health innovations to everyone. General Electric’s MACi ECG [5] and Rice University’s bubble CPAP [6] are examples of devices stripped of superfluous functions that cost from one-half to one-fifteenth that of their average counterparts. These technologies are not simply low-cost versions of medical devices used in richer countries; they are durable, portable, able to function in harsh environments and easy to maintain, with cheap and accessible spare parts [3]. Lean tools developed for low-resource settings are sometimes so cost-efficient that they are better than solutions used in high-income countries. For example, Siemens’ Chinese engineers have developed an inexpensive CT scanner by removing infrequently used settings and options. The resulting machine has cut the cost of treatment by 30 % and has “reversely” spread in the United States [7].

*Opportunistic solutions* refer to the clever use of modern, cheap and available-for-everyone technologies to tackle “old problems”. Mobile phone technologies and the Internet are examples of technologies that can radically change medical possibilities, from improving adherence to antiretroviral treatment via a mobile phone SMS [8] to identifying counterfeit drugs [9]. Another example is how 3D printers may remodel accessibility to medical devices by allowing virtually anyone to manufacture medical tools, from low-cost prosthetics [10] to spare parts of equipment.

*Contextualized adaptations* refer to the diversion of existing techniques, materials or tools for completely different purposes. For example, urinary reagent strips used to evaluate cerebrospinal [11] or synovial fluid [12] were found to be good diagnostic tests, usable in under-resourced environments at virtually no cost. Another example of contextualized adaptation is the Solarclave, a do-it-yourself autoclave made of a bucket containing a pressure cooker and a reflector consisting of 140 small mirrors arranged in a complex geometric way to concentrate and redirect sunrays towards the bucket, heating it up to 120 °C and achieving the physical sterilization standard of the US Centers for Disease Control and Prevention [13]. Surgeons are experts in these adaptations, constantly changing the procedure as a function of the instruments and equipment they have at their disposal. For example, paper clips have been found a (very) cheap and effective alternative to Raney clips in dental surgery [14].

*Local bottom-up innovations* refer to original, simple – and even simplistic – ideas to obtain results not previously attainable. These grassroots innovations often emerge in environments where the scarcity of resources challenges human ingenuity, as shown by the invention of “kangaroo care” for preterm infants [15] or solar disinfection of water to reduce diarrhea in areas where drinking water comes from waterholes not suitable for chemical treatment [16]. Bottom-up innovations frequently grow out of local means and practices; for example, bicycle ambulances are a perfect alternative to car ambulances in places where cars are too costly and not adapted for the traffic density.

## Challenges for frugal innovations

Caregivers in low-resource settings do their best to mimic practices considered optimal despite challenging environments. The creative innovations they develop may not be as effective as those used in high-income settings but often represent alternatives with excellent cost–benefit ratios adapted to their contexts. Of note, these innovations are not and should not be confined to developing countries. For example, accessible low-cost diagnostic tools such as urinary reagent strips for analysis of synovial fluids could be used by physicians in ambulatory care for rapid diagnostic orientation and may avoid the referral of patients to crowded emergency departments. This idea of “reverse innovation” (i.e., the flow of ideas from lower- to higher-income settings) is increasingly garnering attention and has resulted in fruitful partnerships between developed and developing countries [17].

Yet, several challenges remain for frugal innovations.

First, people should remain aware that some bottom-up innovations may be developed on mistaken beliefs and cause more harm than good. For instance, Cola drinks were recommended for rehydration with acute diarrhea for several years before evidence showed that these drinks had low electrolyte content and extremely high osmolality, which may actually worsen diarrhea [18]. Because frugal innovations seek to provide solutions to common healthcare problems, they must be scientifically evaluated before widespread utilization.

Second, frugal innovations may offer effective and cheap solutions to healthcare problems in low-resource settings but may not be adopted. For instance, despite flash heating of breast milk (i.e., heating breast milk by using a glass jar placed in a pot of boiling water) being able to reduce mother-to-child transmission of HIV infection [19], the process is not well implemented in African countries because it requires frequent, unpractical boiling of water and because it indicates that the woman is HIV positive, exposing her to stigma in the community [20]. As with all medical interventions, adoption of medical innovations depends not only on their effectiveness or costs but also on how they can be integrated in patients’ daily lives and/or physician practices.

Finally, many frugal innovations, especially bottom-up innovations, stay local, “below-the-radar” and rarely spread to otherswho might face similar challenges. For instance, a method to perform auto-transfusion when no blood donor is present was developed in South Africa [5], but our discussions with doctors in Democratic Republic of Congo revealed that most of them neither knew nor used this method, which could have saved some patients’ lives.

Examples in this paper represent the tip of the iceberg – just a few of the ingenious practices across the world that have been evaluated and published. Thus, we argue for the creation of a “Compendium of Good Ideas” at http://frugal-innovation-medicine.com, whereby doctors, inventors, patients, and others can share ideas and inventions of frugal innovations for consideration in relevant contexts, scientific evaluation and/or inspiration.

## Conclusion

In constrained environments, where resources are scarce, healthcare providers often craft unexpected solutions to provide adequate healthcare to patients. These inexpensive but effective frugal innovations may be imperfect, but they have the power to improve people’s lives by ensuring that health is within everyone’s reach.
